# Synthesis, Physico-chemical Characterization, Crystal Structure and Influence on Microbial and Tumor Cells of Some Co(II) Complexes with 5,7-Dimethyl-1,2,4-triazolo[1,5-*a*]pyrimidine

**DOI:** 10.3390/molecules22071233

**Published:** 2017-07-22

**Authors:** Luminiţa Măruţescu, Larisa Calu, Mariana Carmen Chifiriuc, Coralia Bleotu, Constantin-Gabriel Daniliuc, Denisa Fălcescu, Crina Maria Kamerzan, Mihaela Badea, Rodica Olar

**Affiliations:** 1 Department of Microbiology, Faculty of Biology, University of Bucharest, 1–3 Aleea Portocalelor Str., 60101 Bucharest, Romania; lumi.marutescu@gmail.com (L.M.); carmen_balotescu@yahoo.com (M.C.C.); crina.saviuc@yahoo.com (C.M.K.); 2 Environment and Earth Sciences Department, Research Institute of the University of Bucharest—ICUB, Life, Spl. Independentei 91–95, 010271 Bucharest, Romania; 3 Department of Inorganic Chemistry, Faculty of Chemistry, University of Bucharest, 90–92 Panduri Str., 050663 Bucharest, Romania; larisa.calu@yahoo.com (L.C.); denisa_ktalina_89@yahoo.com (D.F.); 4 Stefan S Nicolau Institute of Virology, 285 Mihai Bravu Ave., 030304 Bucharest, Romania; cbleotu@yahoo.com; 5 Organisch-Chemisches Institut, Westfälische Wilhelms-Universität Münster, Corrensstrasse 40, 48149 Münster, Germany; constantin.daniliuc@uni-muenster.de

**Keywords:** Co(II) complex, 1,2,4-triazolo[1,5-*a*]pyrimidine, *cis*-disposition, biofilm, cytotoxicity

## Abstract

Three complexes, namely [Co(dmtp)_2_(OH_2_)_4_][CoCl_4_] (**1**), [Co(dmtp)_2_Cl_2_] (**2**) and [Co(dmtp)_2_(OH_2_)_4_]Cl_2_∙2H_2_O (**3**) (dmtp: 5,7-dimethyl-1,2,4-triazolo[1,5-*a*]pyrimidine), were synthesized and characterized by spectral (IR, UV-Vis-NIR), and magnetic measurements at room temperature, as well as single crystal X-ray diffraction. Complex (**1**) crystallizes in monoclinic system (space group *C*2/c), complex (**2**) adopts an orthorhombic system (space group *P*bca), and complex (**3**) crystallizes in triclinic system (space group P$\bar{1}$). Various types of extended hydrogen bonds and π–π interactions provide a supramolecular architecture for all complexes. All species were evaluated for antimicrobial activity towards planktonic and biofilm-embedded microbial cells and influence on HEp-2 cell viability, cellular cycle and gene expression.

## 1. Introduction

The rapid selection of resistance after antibiotic and cytostatic drug treatment represents an emerging and acute health problem at a global level. Furthermore, many pathogenic microorganisms have the ability to adhere, colonize and develop biofilms on prosthetic materials and natural tissues, exhibiting an increased resistance, also called tolerance, to different stress factors, including host defense mechanisms and antibiotics, as compared with their planktonic counterparts, being responsible of a wide range of human chronic, persistent and hard to treat infections [1,2,3]. In order to overcome this challenging problem of increasing antimicrobial resistance, there is an urgent need to design new antimicrobials with better activity profiles and lower toxicity.

Derivatives bearing [1,2,4]-triazolo[1,5-*a*]pyrimidine fused ring systems have been widely investigated for the design of new drugs in view of the similarity of their chemical structure with that of the purine nucleobases, i.e., adenine and guanine. Such derivatives are interesting from both biological and chemical perspectives. Several methoxynaphthalen- [4] and thieno[2,3-*d*][1,2,4]-triazolo[1,5-*a*]pyrimidines [5] have been shown to exhibit anti-inflammatory activity, while 7-methyl-5-phenyl-4,5-dihydro-[1,2,4]triazolo[1,5-*a*] pyrimidine demonstrated both analgesic and anti-inflammatory effects [6]. Some triazolopyrimidines linked with a disubstituted pyrazole ring showed promising anti-tuberculostatic activity [7], while derivatives substituted with trifluoromethyl, aryl and aliphatic amines exhibited good activity against chloroquine-resistant *Plasmodium falciparum* [8]. 

Species of the [1,2,4]triazolo[1,5-*a*]pyrimidine-5,7-disubstituted type with aromatic bulky groups have proven antiviral potency against wild-type and resistant HIV-1 strains [9] and a series of 5-chloro-6-(trifluorophenyl)-*N*-fluoroalkyl [1,2,4]triazolo[1,5-*a*]pyrimidines exhibited antitumor activity, both in vitro and in vivo, affecting tubulin polymerization [10]. Disubstituted-[1,2,4]-triazolo[1,5-*a*]pyrimidine species showed anticonvulsant effects, [11] while 2-(alkylthio)-[1,2,4]-triazolo[1,5-*a*]pyrimidines have therapeutic potential for cardiovascular diseases [12].

In addition, the complexes with triazolopyrimidine derivatives were investigated for their antimicrobial [13,14,15], antitumor [16,17,18,19,20,21,22,23,24,25] and antiparasitic [26,27] features. Among these, organotin(IV) complexes with disubstituted [1,2,4]triazolo[1,5-*a*]pyrimidines showed in vitro efficiency against Gram-positive bacteria [13], such as staphylococcal strains [14]. Antitumor activity was evidenced for Ag(I) [16], Sn(IV) [17], Ru(II) [18], Pt(IV) [19] and Pt(II) [20,21,22,23,24,25] complexes with a large diversity of unsubstituted [18,19], mono- [19], di- [17,19,20,22,23,24,25] or trisubstituted [16,18,21] [1,2,4]triazolo[1,5-*a*]pyrimidines as ligands.

The complexes with 5,7-dimethyl-1,2,4-triazolo[1,5-*a*]pyrimidine (dmtp) as ligand have been extensively characterized from the physico-chemical point of view, but only few studies concerning their biological properties have been reported [15,20,22,26,27,28]. A moderate activity against acute promyelocytic leukemia, rectal adenocarcinoma and mouse bladder tumor cells has been reported for a platinum (IV) complex and activity superior to that of cisplatin on B16 melanoma cell lines for Pt(II) species with dmtp and hexafluoroglutarate [20]. Furthermore, the complex [Pt(mal)(dmtp)_2_]∙4H_2_O (mal=malonate) exhibited also a moderate antiproliferative activity against the human T47D cell line (cisplatin-resistant human ductal breast epithelial tumor cell line), A549 (lung adenocarcinoma epithelial cell line), and the 4T1 cell line (mouse breast tumor model) [22]. Some cobalt(II) complexes with dmtp exhibit good antimicrobial activity against *Micrococcus* sp., *Staphylococcus* sp. and *Proteus* sp. strains [15]. In vitro and in vivo studies (murine model) revealed a strong antiparasitic potential of some Mn(II), Fe(II), Co(II), Ni(II), Cu(II) and Zn(II) complexes with dmtp against *Leishmania* spp. (*Leishmania infantum*, *Leishmania braziliensis*) and *Trypanosoma cruzi* [26,27].

In this work we aimed to combine the chemistry of Co(II) with that of 1,2,4-triazolo[1,5-*a*]pyrimidine moiety in an attempt to design novel metal-based biologically active candidates for antimicrobial therapy, that could be efficient both against planktonic and biofilm-embedded microbial cells. The compounds’ cytotoxicity was evaluated on the HEp-2 tumor cell line, together with their influence on the expression of a gene involved in cellular transport.

## 2. Results and Discussion

### 2.1. Synthesis of the Complexes

**Scheme 1:**
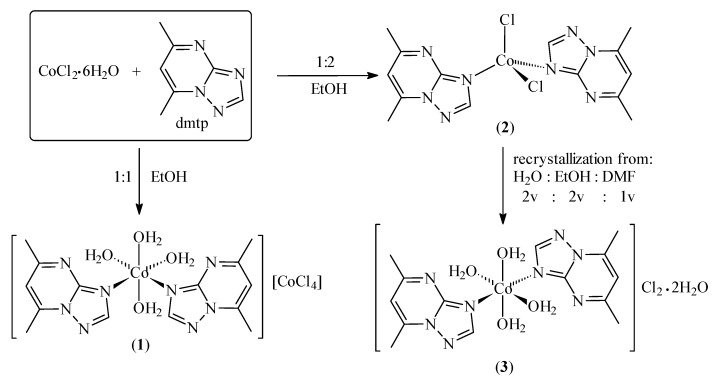
The synthesis routes of the complexes.

The 5,7-dimethyl-1,2,4-triazolo[1,5-*a*]pyrimidine (dmtp) ligand produces the complexes [Co(dmtp)_2_(OH_2_)_4_][CoCl_4_] (**1**) and [Co(dmtp)_2_Cl_2_] (**2**) in its reactions with cobalt(II) chloride in 1:1 and 1:2 molar ratio, respectively. The compound [Co(dmtp)_2_(OH_2_)_4_]Cl_2_∙2H_2_O (**3**) was obtained from the recrystallization of compound **2** from a mixture of water containing solvents, as depicted in Scheme 1.

### 2.2. Characterization of the Complexes

#### 2.2.1. Description of the X-ray Crystal Structures of the Complexes

**Figure 1:**
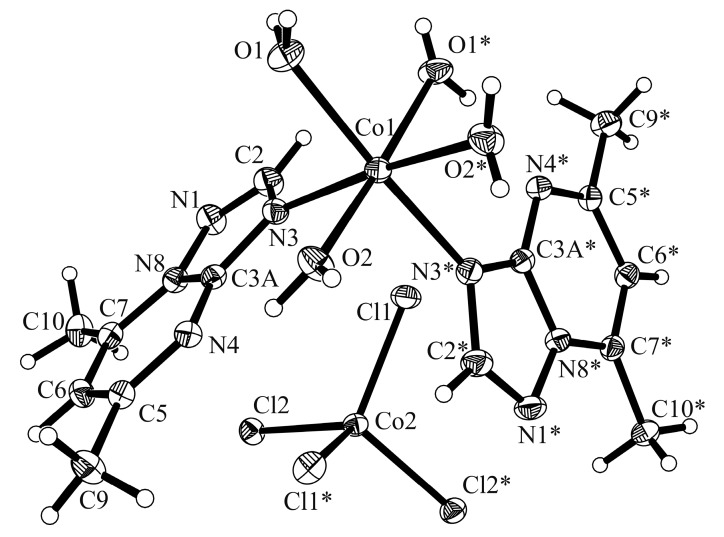
Crystal structure of [Co(dmtp)_2_(OH_2_)_4_][CoCl_4_] (**1**) with the labeling scheme for the non-hydrogen atoms. Symmetry transformations used to generate equivalent atoms marked with *: −x + 1, y, −z + 1/2. Thermal ellipsoids represent 30% probability levels.

The crystal parameters, data collection, and refinement details of the complexes are summarized in Table 1. The complex [Co(dmtp)_2_(OH_2_)_4_][CoCl_4_] (**1**) crystallizes in the monoclinic *C*2/c space group and its crystal structure consists of discrete [Co(dmtp)_2_(OH_2_)_4_]^2+^ and [CoCl_4_]^2−^ units as shown in Figure 1.

Both metal atoms Co1 and Co2 are located on a crystallographic two-fold axis. The Co1 atom presents a distorted octahedral geometry being surrounded by four water and two dmtp molecules in a *cis* arrangement, which is unique among the [M(dmtp)_2_(OH_2_)_4_]^2+^ species [15,29]. The organic ligand is coordinated in a unidentate fashion through N3 atom as observed for other complexes [15,29]. The bonds length and angles are similar with those reported for the free ligand [30] except for the N3–C2 bond that becomes slightly longer (1.356(3) Å over 1.337(3) Å) in complex as result of nitrogen atom coordination (Table S1, Supplementary Materials).

The Co1-N bond lengths of 2.154(2) Å are in the range observed for [Co(dmtp)_2_(H_2_O)_4_]Br_2_∙2H_2_O [15] and for [Co(dmtp)_2_(H_2_O)_4_](NO_3_)_2_ [29] species containing the same complex cation. The Co1–O bond lengths fluctuate between 2.085(2) and 2.113(2) Å indicating that the water molecules are involved in additional interactions. The tetrahedral Co2 ion is surrounded by four chloride ions with Cl–Co2–Cl angles varying from 108.3(1) to 113.6(1)° and Co2–Cl bonds lengths ranging from 2.276(1) to 2.278(1) Å (Table S1, Supplementary Materials).

**Figure 2:**
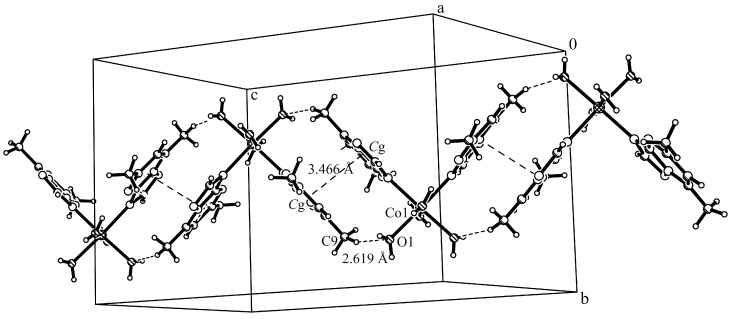
Excerpt of packing diagram of complex (**1**) showing the formation of zig-zag chains along the c axis involving the cationic units through π**^…^**π stacking interactions and additionally C–H**^...^**O hydrogen bonds.

**Figure 3:**
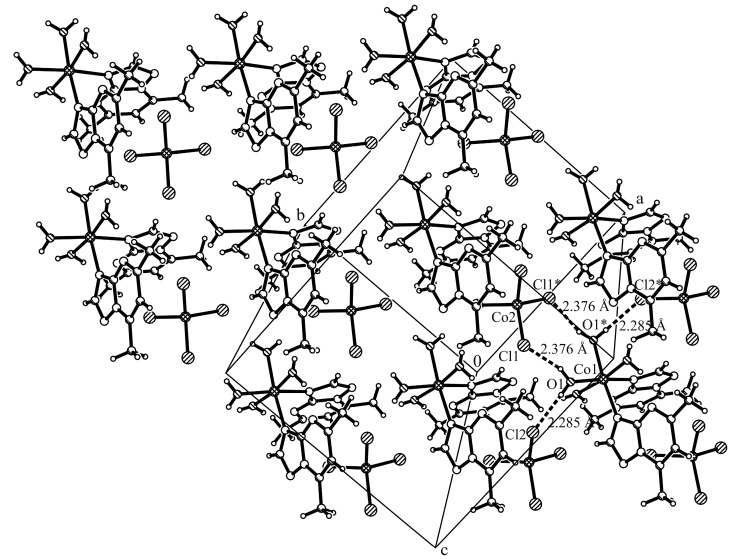
Packing diagram of complex (**1**) with view perpendicular to *xy*-plane presenting the O–H**^...^**Cl interactions between the cation and anion.

The complex cations present the formation of zig-zag chains along the *c* axis through π**^…^**π stacking interactions involving the dmtp ligands (the corresponding centroid-centroid distance between the pyrimidine aromatic rings is *C*g**^…^***C*g 3.466 Å, where the *C*g is the centroid of atoms C3a/N4/C5/C6/C7/N8 and the shortest distance between the dmtp ligands is 3.455 Å) and through additionally C–H**^…^**O interactions (C9–H9C**^…^**O1 2.619(3) Å), which are therefore responsible for *cis* disposition of dmtp units (Figure 2, Table S2, Supplementary Materials). The packing is completed through the hydrogen bonds formed with the complex anions. Two coordinated water molecules are involved in intramolecular interactions with the nitrogen atoms from dmtp ligand (O2–H03**^…^**N4, 1.887(2) Å), while the other two link together the cations and anions through O–H**^…^**Cl interactions (O1–H01**^…^**Cl2, 2.285(3) Å; O1–H02**^…^**Cl1, 2.376(2) Å (Figure 3).

**Figure 4:**
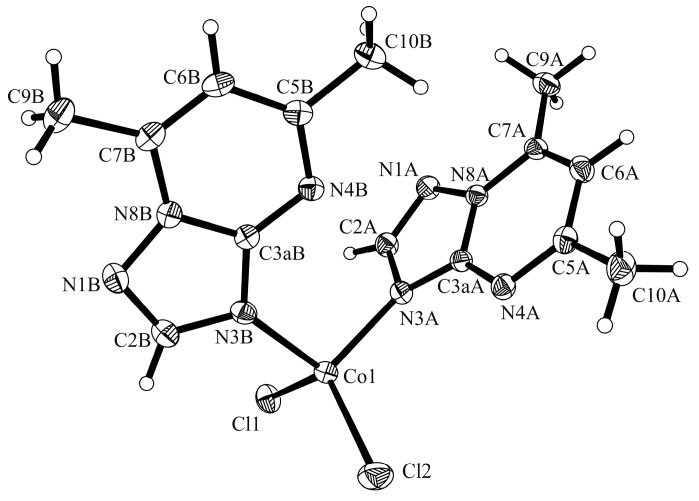
Crystal structure of [Co(dmtp)_2_Cl_2_] (**2**) with the labeling scheme for the non-hydrogen atoms. Thermal ellipsoids represent 30% probability levels.

Complex [Co(dmtp)_2_Cl_2_] (**2**) crystallizes in the orthorhombic *P*bca space group and the Co1 atom adopts a tetrahedral coordination environment by linking to two chloride anions and two triazolopyrimidine molecules through the N3 atom (Figure 4). The Co1–N3A and Co1–N3B bond lengths are shorter in comparison with those observed for octahedral Co(II) compounds [15,29]. The C2A–N3A and C2B–N3B bond lengths are longer in comparison with the free ligand as result of the nitrogen atom coordination (Table S3, Supplementary Materials).

**Figure 5:**
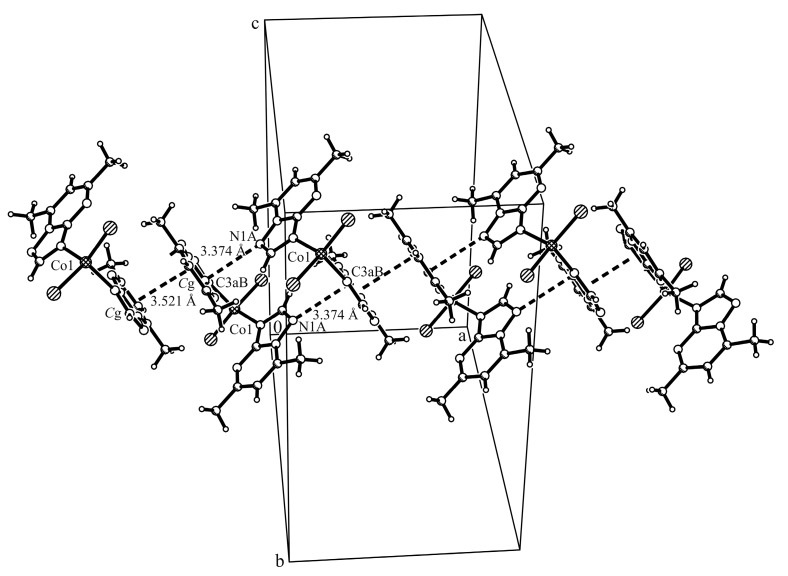
Excerpt of packing diagram of complex (**2**) presenting π**^…^**π interactions between the dmtp ligands (Symmetry codes: #1: −x, −y + 1, −z + 1; #2: −x + 1, −y + 1, −z + 1).

The formation of linear chains along the *x*-axis containing supramolecular dimers formed through π**^…^**π stacking interactions between the dmtp ligands (centroid-centroid distance between the pyrimidine aromatic rings is *C*g**^…^***C*g 3.521 Å, where the *C*g is the centroid of atoms C3aB/N4B/C5B/C6B/C7B/N8B; shortest distance between the pyrimidine rings is C3aB**^…^**C7B 3.374 Å) and forward interactions between these dimers involving the dmtp ligands and the nitrogen atom from the neighboring of triazole ring (C3aB**^…^**N1A 3.158 Å) were observed in the packing diagram (Figure 5, Table S3, Supplementary Materials).

**Figure 6:**
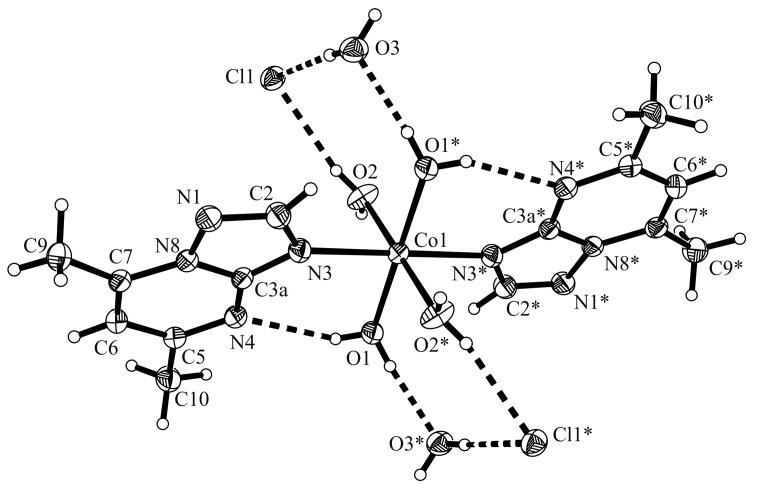
Crystal structure of [Co(dmtp)_2_(OH_2_)_4_]Cl_2_·2H_2_O (**3**) with the labeling scheme for the non-hydrogen atoms. Symmetry transformations used to generate equivalent atoms marked with *: −x − 1, −y, −z. Thermal ellipsoids represent 30% probability levels.

The crystals of compound [Co(dmtp)_2_(OH_2_)_4_]Cl_2_·2H_2_O (**3**) were isolated from a mixture of DMF:C_2_H_5_OH:H_2_O containing complex (**2**). This species crystallizes in the triclinic P$\bar{1}$ space group and the structure consists of discrete cations [Co(dmtp)_2_(H_2_O)_4_]^2+^, two crystallization water molecules and two chloride anions (Figure 6). The Co1 metal ion is located in the inversion center and exhibits a distorted octahedral coordination environment assured by four water molecules and two nitrogen atoms N3 from triazolopyrimidine units in a *trans* arrangement. 

**Figure 7:**
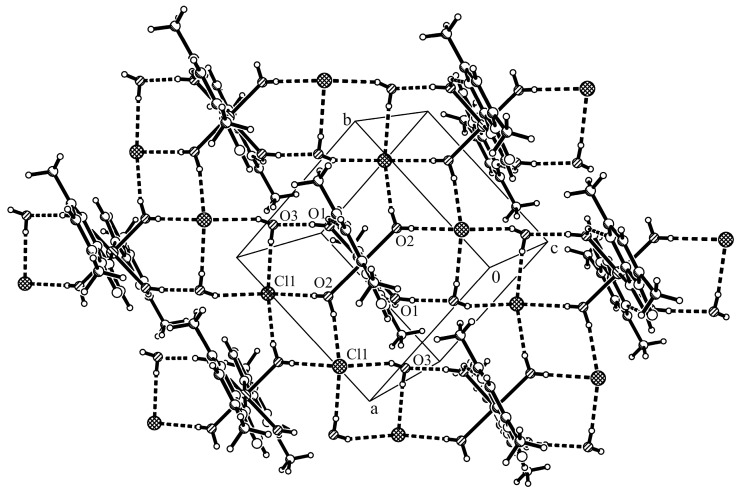
Packing diagram of complex [Co(dmtp)_2_(OH_2_)_4_]Cl_2_·2H_2_O (**3**) viewed perpendicular to the *xy*-plane. Thick dashed lines represent O–H···Cl and O–H···O interactions.

The Co1-O bond lengths (2.059(3) and 2.096(2) Å) indicate that coordinated oxygen atoms are involved in different interactions with chloride anions and crystallization water molecules, respectively (Table S4). The packing diagram of this complex presents chlorine-water chain along *b* axis similar with that observed for corresponding bromine complex [15]. The chlorine atom is surrounded by four water molecule in a pseudotetrahedral geometry. The complex cations are assembled through additional week π**^…^**π stacking interactions (centroid-centroid distances between the pyrimidine aromatic rings are *C*g**^…^***C*g 3.70 Å; see Figure 7, Table S5, Supplementary Materials). Intramolecular hydrogen bonds between one coordinated water molecule and the pyrimidine nitrogen atom (O1–H1B**^…^**N4 2.200 Å) were observed.

The tetragonality parameter T quantifies the Jahn Teller distortion and is defined as R_in_/R_out_, where R_in_ represents the mean of the in-plane Cu–N or Cu–O bond lengths and R_out_ represents the average of the corresponding axial bond lengths [31]. The obtained values of 0.99 for complex (**1**) and 0.97 for complex (**3**) indicates a slightly elongated octahedral environment around Co(II) for the last species, as is expected for a complex with both Jahn Teller effect and mixed ligands in a *trans* disposition.

#### 2.2.2. Infrared Spectra

The IR spectra of the complexes display bands in the 1624–1635 and 1038–1045 cm^−1^ ranges, respectively, due to the triazole precursor (Table 2, Figure S1). The bands located in the 3100–3133, 1550–1555 and 1426–1465 cm^−1^ ranges represent instead a pattern for the pyrimidine ring [26]. In the IR spectrum of the dmtp the intense band at 1635 cm^−1^ is assigned to the stretching vibration ν(C=N) of triazole ring. This band is shifted by 9–11 cm^−1^ toward lower wavenumbers in complexes spectra as an indicative of ligand coordination through a nitrogen atom of this ring. 

In the characteristic ranges for water, a broad band assigned to ν(OH) stretching vibration can be observed in the range 3407–3415 cm^−1^, except for complex **2**. Additional bands in the 662–779 cm^−1^ range, characteristic for coordinated water molecules, can be observed for complexes **1** and **3** [31]. Around 490 and 535 cm^−1^ two supplementary bands assigned to stretching vibrations ν(Co–N) and ν(Co–O), appear in the spectra of complexes except for complex **2** [32].

#### 2.2.3. UV-Vis-NIR Spectra and Magnetic Moments

UV-Vis-NIR spectra together with magnetic moments at room temperature provide useful information concerning the oxidation state of cobalt, stereochemistry, as well as the ligand field strength. Three bands can be noticed in the UV region of the ligand spectrum assigned to π→π* transition arising from both C=N groups of pyrimidine and triazole moieties, respectively (Table 3, Figure S2, Supplementary Materials). These bands are overlapped and shifted in the complexes’ spectra as a result of coordination.

The spectrum of complex **1** exhibits the pattern of two Co(II) coordination environments, octahedral and tetrahedral, respectively. Thus, the low intensity bands that appear at 8970 and 19,400 cm^−1^ are assigned to spin allowed d-d transitions for an octahedral stereochemistry. The bands assigned to tetrahedral components are split as result of both distortion and spin orbit coupling [33]. The value of magnetic moment at the room temperature for Co(II) complex is 4.47 B.M. per metallic ion, in accordance with the presence of both octahedral and tetrahedral Co(II) coordination environment, the first one being responsible of the small orbital contribution [34].

The spectrum of compound **2** is characteristic of a distorted tetrahedral Co(II) coordination environment revealed by the two intense bands in visible and near-infrared regions, assigned to the spin allowed transitions ^4^A_2_→^4^T_1_(F) and ^4^A_2_→^4^T_1_(P). Both bands are split in two components as a result of lower symmetry generated by the different nature of donor atoms. The magnetic moment of 4.29 B.M. lies in the range accepted for this stereochemistry.

The low intensity and the position of the bands in the spectrum of compound **3** indicate an octahedral Co(II) coordination environment, furthermore confirmed by the increased magnetic moment value of 5.00 B.M. as a result of orbital contribution.

### 2.3. Biological Activity

#### 2.3.1. Antimicrobial Activity

The antimicrobial activity of the compounds was assayed against reference and clinical Gram-negative and Gram-positive bacteria as well as fungal strains. It is to be pointed out that the clinical strains exhibited a multidrug resistance phenotype, defined as acquired non-susceptibility to at least one agent from three or more antimicrobial categories. Some of the tested bacterial strains belong to species in the top ten of the most frequently encountered agents responsible of hospital infections in entire world, the so-called ESKAPE pathogens (i.e., *Staphylococcus aureus*, *Pseudomonas aeruginosa* and *Klebsiella pneumoniae*) [35,36,37]. Beside bacterial infections, fungal infections caused especially by *Candida* sp. are also a growing problem particularly in severely immune-compromised patients [36]. Having in view the differences in the physiology and susceptibility to antibiotics of biofilm-embedded microorganisms, the antimicrobial activity of the obtained complexes was investigated on planktonic and adherent cells grown in biofilm developed in plastic wells.

**Figure 8:**
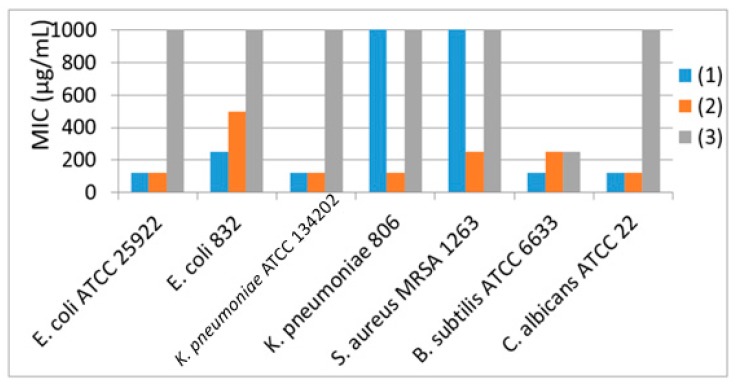
Minimum inhibitory concentration (µg mL^−1^) of the complexes.

Complexes **1** and **2** revealed lower MIC values, in comparison with **3** (Figure 8). Complexes **1** and **2** exhibited a good activity expressed by a MIC value of 125 μg mL^−1^ against *E. coli* ATCC 25922, *K. pneumoniae* ATCC 134202 and *C. albicans* ATCC 22 strains. Furthermore, complex **1** exhibited a good activity also against *B. subtilis* ATCC 6633, and complex **2** against *K. pneumoniae* 806.

Complex **1** also exhibited an inhibitory effect upon the adherence ability of *E. coli* ATCC 25922, *E. coli* 832, *B. subtilis* ATCC 6633 and *C. albicans* ATCC 22 strains up to a concentration of 62.50 μg mL^−1^ (Table 4). Complex **2** inhibited the adherence ability of all tested strains with minimal biofilm eradication concentration (MBEC) values generally ranging from 62.50 to 125 μg mL^−1^. It is worth mention that the anti-biofilm activity of the obtained complexes was preserved even at subinhibitory concentrations.

These results indicate that complex **2** is the most efficient agent against the planktonic cells, exhibiting the largest spectrum of antimicrobial activity, being also the most active anti-biofilm compound. The antimicrobial activity spectrum and intensity of the tested complexes may be assigned either to variations in the microbial cell wall permeability or to different target molecules.

**Figure 9:**
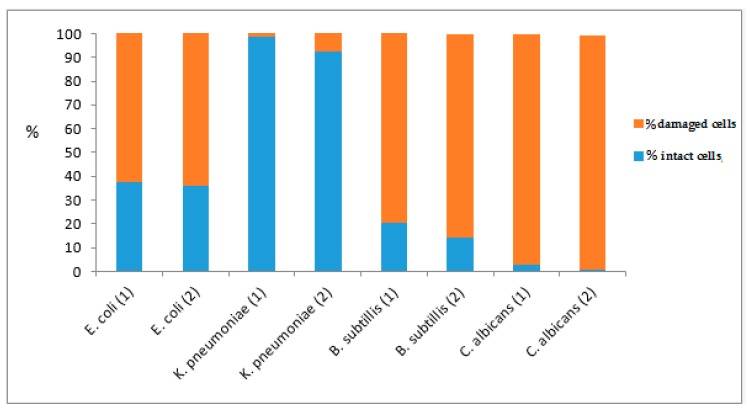
The effect of complexes **1** and **2** on cellular membrane permeability assessed by flow cytometry.

The possible mechanisms of the antimicrobial activity for the most active compounds **1** and **2** (i.e., MBEC of 52.50 μg mL^−1^) have been tested by flow cytometry analysis. Also, both compounds induced changes in the cellular membranes permeability as revealed by the increased fluorescence signal of the PI, with a higher activity for complex **2** (Figure 9). The most significant membrane lesions were induced in the Gram-positive bacterial and fungal strains, probably due to the fact that in the Gram-negative bacteria, the presence of the outer membrane and of polysaccharide capsular material are protecting the cellular wall from the accumulation of the tested compounds. 

**Figure 10:**
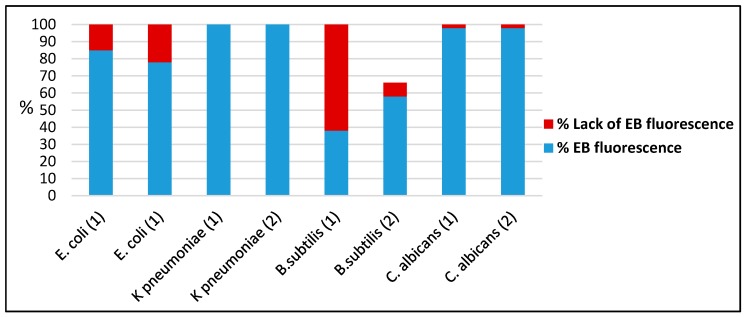
The effect of complexes **1** and **2** on the activity of efflux pumps assessed by flow cytometry.

The flow cytometric measurements of EB fluorescence revealed that the exposure of microbial cells to the compounds **1** and **2** did not cause a significant increase of permeability for this dye, indicating that the microbial efflux pumps activity was not affected. An exception was the compound (**1**) which exhibited a good efflux pump inhibitory activity against the *B. subtilis* strain (Figure 10) demonstrated by increased level of EB fluorescence as compared to the viable control cells.

Therefore, the flow cytometry analysis revealed that the main mechanism of the antimicrobial activity of complexes is represented by the induction of lesions at the level of cellular membranes affecting their selective permeability, and in lesser extent, by inhibiting the expression of efflux pumps.

#### 2.3.2. In Vitro Assessment of the Complexes Influence on the HEp-2 Cellular Cycle and Gene Expression

The assay of compounds influence on mammalian cells has drawn considerable attention having in view their potential biomedical applications. The complexes of essential elements like cobalt are generally less toxic than those of nonessential ones. However, the ability of cobalt(II) ion to both generate complexes and initiate redox reactions could induce an efficient interaction with biomolecules. The flow cytometry analysis of the HEp-2 cellular cycle in the presence of complexes evidenced the occurrence of a sub-G1 peak that indicates the occurrence of apoptotic cells. This aspect correlated with the data obtained by using Annexin V FITC-PI kit, revealing that the complexes **2** and **3** induced a drastic decrease in HEp-2 cell viability (Table 5).

The pro-apoptotic effect of cobalt complexes on tumor cells could open a new perspective for the use of these compounds as anti-tumor agents [38,39,40,41,42]. The design of new metal-based anticancer drugs is taken into consideration in order to enhance the selectivity through various non-covalent binding interactions such as intercalation or electrostatic binding, so the cobalt(II) complexes with dmtp could exhibit an anticancer potential considering the redox-activity of Co(II) that may damage DNA by inducing oxidative stress and/or producing DNA modifications by coordination while the ligand could both intercalate and generate hydrogen bonds with nucleotides.

**Figure 11:**
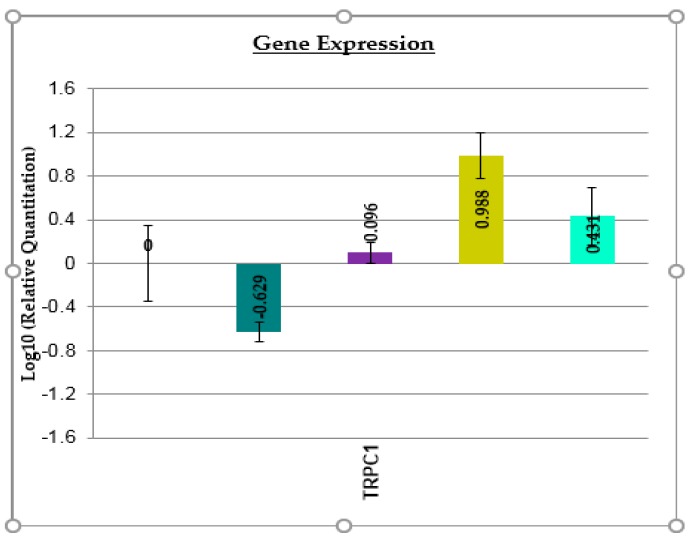
The effects of the tested compounds (100 µg mL^−1^ solution) on the expression of TRPC1 gene (dmtp, dark green; (1), magenta; (2), olive; (3), light green).

In order to elucidate the role of the complexes on the HEp-2 cells, the expression of transient receptor potential channel 1 (TRPC1) was investigated by real time PCR. All tested complexes increased the levels of TRPC1 (Figure 11), a nonspecific cation channel located in the plasma membrane of numerous cell types, suggesting that the interference with Ca^2+^ transport could be a possible mechanism of their pro-apoptotic effect on HEp-2 cells, also explaining their antimicrobial, and particularly, the anti-biofilm activity.

It is already known that the polysaccharide biofilm matrix cross-links with bivalent cations, particularly Ca^2+^, Mg^2+^, but also with toxic metal cations, stabilizing the biofilm structure, but also enhancing the attachment of planktonic cells to a certain surface. Therefore, one of the most promising strategies for biofilms dispersal is the use of cations chelating agents, which could weaken the biofilm matrix structure, rendering the biofilm embedded cells susceptible to host immune effectors or antibiotics. The bivalent cations, such as Ca^2+^ also stabilize and reinforce the microbial cell wall, therefore, the chelation of these cations could sensitize the microbial wall and increase the permeability for antimicrobial agents.

## 3. Experimental Section

### 3.1. General Information

High purity reagents purchased from Sigma-Aldrich (Saint-Louis, MO, USA, CoCl_2_∙6H_2_O), Merck (Darmstadt, Germany 2,4-pentanedione) and Fluka (Saint-Louis, MO, USA, 3-amino-4H-1,2,4-triazole) were used as received without further purification. The 5,7-dimethyl-1,2,4-triazolo[1,5-*a*]pyrimidine (dmtp) was obtained by condensation of 2,4-pentanedione and 3-amino-4H-1,2,4-triazole, purified by ethanol recrystallization and the purity was checked by thin layer chromatography.

Chemical analysis of carbon, nitrogen and hydrogen has been performed using a PE 2400 analyzer (Perkin Elmer, Waltham, MA, USA). IR spectra were recorded in KBr pellets with a Tensor 37 spectrometer (Bruker, Billerica, MA, USA) in the range 400–4000 cm^−1^. Electronic spectra by diffuse reflectance technique, with Spectralon as standard, were recorded in the range 200–1500 nm, on a V670 spectrophotometer (Jasco, Easton, MD, USA). Magnetic measurements were done at room temperature, on a fully integrated Vibrating Sample Magnetometer (VSM) system 7404 (Lake Shore, Westerville, OH, USA), calibrated with a Ni-0.126 g sphere-SRM 772a. The VSM was calibrated with Hg[Co(NCS)_4_] as standard. The molar magnetic susceptibilities were calculated and corrected for the atomic diamagnetism. Powder X-ray diffraction (XRD) patterns were recorded using an XRD-7000 diffractometer (Shimadzu, Kyoto, Japan) with Cu Kα radiation (λ = 1.5406 Å, 40 kV, 40 mA) at a step of 0.2° and a scanning speed of 2 degrees min^−1^ in the 5–60 degrees 2θ range.

### 3.2. Synthesis of Complexes

#### 3.2.1. [Co_2_(dmtp)_2_(OH_2_)_4_][CoCl_4_] (**1**)

To a solution containing cobalt(II) chloride hexahydrate (1.19 g, 5 mmol) in ethanol (25 mL) was added a solution containing dmtp (0.74 g, 5 mmol) in ethanol (25 mL). The reaction mixture was magnetically stirred at 50 °C for 2 h, until a sparingly soluble species was formed. The precipitate was filtered off, washed several times with cold ethanol and air-dried. Crystals suitable for X-ray analysis were obtained by slow evaporation of complex solution in ethanol. Yield: 86% (1.27 g), Anal. Calc.: C, 26.67; H, 3.65; N, 17.92; Found: C, 26.77; H, 3.85; N, 17.84%.

#### 3.2.2. [Co(dmtp)_2_Cl_2_] (**2**)

To a solution containing cobalt(II) chloride hexahydrate (1.19 g, 5 mmol) in ethanol (25 mL) was added dmtp (1.48 g, 10 mmol) in ethanol (50 mL). The reaction mixture was magnetically stirred at 50 °C for 1 h, until a blue colored species was formed. The solution was left to stand at room temperature for one week while blue crystals arose from the solution. The complex synthesis by another method was already reported [43]. Yield: 79% (1.58 g), Anal. Calc.: C, 39.46; H, 3.78; N, 26.29; Found: C, 39.51; H, 3.71; N, 26.31%.

#### 3.2.3. [Co(dmtp)_2_(OH_2_)_4_]Cl_2_·2H_2_O (**3**)

Complex **3** was obtained by recrystallization of complex **2** (1.20 g) from a DMF:C_2_H_5_OH:H_2_O solution (1:2:2 *v:v:v*). The complex synthesis by another method was already reported [43]. Yield: 60% (0.90 g), Anal. Calc.: C, 31.47; H, 5.28; N, 20.97, Found: C, 31.49; H, 5.22; N, 20.98%.

### 3.3. X-ray Crystallography

Data sets were collected with a Nonius Kappa CCD diffractometer (Nonius B. V., Delft, The Netherlands). Programs used: data collection, COLLECT [44]; data reduction Denzo-SMN [45]; absorption correction, Denzo [46]; structure solution SHELXS-97 [47]; structure refinement SHELXL-97 [48] and graphics, XP [49], R-values are given for observed reflections, and wR^2^ values are given for all reflections. The crystallographic data for (**1**)–(**3**) were deposited at Cambridge Crystallographic Data Centre as supplementary publication CCDC Nos. 944825, 1031208, 1031209. These data can be obtained free of charge via http://www.ccdc.cam.ac.uk/data_request/cif, or by e-mailing data_request@ccdc.cam.ac.uk, or by contacting the Cambridge Crystallographic Data Centre, 12 Union Road, Cambridge CB2 1EZ, UK; Fax: +44-1223-336033. The powder diffraction patterns for all complexes (Figure S3, Supplementary Materials) matched well with that simulated from single crystal structure data indicating that bulk samples were isolated as pure phases.

### 3.4. Biological Assays

#### 3.4.1. Screening of the Antimicrobial Properties

The antimicrobial activity of the complexes was determined against reference and clinical strains, i.e., Gram-negative: *Escherichia coli* ATCC 25922*, Escherichia coli* 832 (positive urine culture), *Klebsiella pneumonia* ATCC 134202*, Klebsiella pneumonia* 806 (wound secretion infection) and Gram-positive: methicillin resistant *Staphylococcus aureus* (MRSA) 1263 (wound secretion infection)*, Bacillus subtilis* ATCC 6633 and the fungal strain *Candida albicans* ATCC 22.

Microbial suspensions of 1.5 × 10^8^ CFU mL^−1^ (0.5 McFarland density) obtained from 15–18 h bacterial cultures developed on solid media were used. The antimicrobial activity was tested on Muller Hinton agar medium. The compounds (ligand, cobalt(II) chloride and complexes) were dissolved in DMSO and the starting stock solution was of 1000 μg mL^−1^ concentration. The qualitative screening was performed by an adapted diffusion method as previously reported [50].

The quantitative assay of the antimicrobial activity was performed by the liquid medium microdilution method, in 96 multi-well plates, in order to establish the minimal inhibitory concentration (MIC) value. In this purpose, serial two-fold dilutions of the compounds ranging between 1000 and 1.95 µg mL^−1^ were performed in a 200 μL volume of broth and each well was seeded with 20 μL microbial inoculum. Sterility control (wells containing only culture medium) and culture controls (wells containing culture medium seeded with the microbial inoculum) were used. The influence of the DMSO solvent was also quantified in a series of wells containing DMSO, diluted accordingly with the dilution scheme used for the complexes. The plates were incubated for 24 h at 37 °C, and MIC values were considered as the lowest concentration of the tested compound that inhibited the visible growth of the microbial overnight cultures [50].

The assessment of the complexes influence on the microbial ability to colonize an inert substratum with the establishment of the minimal biofilm eradication concentration (MBEC) was performed by the micro-titer method, following previously described protocols [50]. The absorbance at 490 nm was measured with an Apollo LB 911 ELISA reader (Berthold Technologies GmbH & Co. KG, Bad Wildbad, Germany). All biological experiments were performed in triplicates.

Flow cytometry was carried out in order to evaluate the influence of the tested compounds on cellular membranes permeability and the microbial efflux pump activity, using two DNA intercalator fluorochromes, i.e., propidium iodide (PI, 10 µg mL^−1^) and ethidium bromide (EB, 5 µg mL^−1^). PI was used for the cellullar viability determination, as living cells are impermeable to this dye and EB was used for the assessment of the bacterial efflux pumps activity. EB passes across the cytoplasmic membrane and accumulates inside the bacterial cell. Efflux pumps of bacteria recognize this substrate and are able to extrude it into the medium. Staining procedures were applied to the harvested cells grown in the presence of the tested compounds at the concentration MIC/2. The cells were centrifuged at 13,000 rpm for 3 min, washed 2 times, resuspended in PBS, stained with 10 μL PI or EB and incubated for 10 min at 4 °C in the dark. Heat-treated cells for 30 min at 100 °C were used as positive controls and viable cells were used as negative controls. The samples were analyzed with a FACS Calibur instrument (Becton, Dickinson and Company, San Jose, CA, USA) equipped with a 488 nm argon laser, using 670 nm long pass filter for the samples stained with PI and (585 ± 42) nm band pass filter for the samples stained with EB. The log scale was used for all the measured parameters and the photomultiplier tube voltage were SSC (side scatter) −550 V, 670 nm long pass filter −480 V and 585 ± 42 nm band pass filter −500 V. 10,000 events were collected in all runs.

#### 3.4.2. Mammalian Cells Morphology and Apoptosis Evaluation

HEp-2 cell line (CCL 223™) was cultivated in RPMI 1640 (Gibco, Grand Island, NY, USA) supplemented with 10% heat-inactivated bovine serum and penicillin/streptomycin at 37 °C with 5% CO_2_. 

To prepare cells for plating into 24-well plate, adherent cells were detached, centrifuged, suspended in fresh medium, counted by trypan blue exclusion and adjusted to 5 × 10^4^ cells mL^−1^. Cells were cultivated for 24 h at 37 °C, in 5% CO_2_ prior to addition of compounds. The substances were initially dissolved into DMSO, and added to the cell monolayer to a final concentration of 100 µg mL^−1^. The cell morphology was evaluated using an inverted microscope. 

In order to discriminate between intact and treatment affected cells, the Annexin V-FITC Apoptosis Detection Kit I (BD Bioscience Pharmingen, CA, USA) was used, according to manufacturer protocol. Briefly, the total cells were suspended in 100 µL of binding buffer (10 mM of HEPES/NaOH, pH value 7.4, 140 mM NaCl, and 2.5 mM CaCl_2_), and stained with 5 µL Annexin V-FITC and 5 µL PI for 10 min in dark prior to analyze using an Epics flow cytometer (Beckman Coulter, IN, USA). At least 10,000 events from each sample were acquired. The percentage of cells affected by the treatment was determined using the FlowJo software [51].

A number of 5 × 10^5^ HEp-2 cells/well were plated in 6-well plate and treated for 24 h with 100 µg mL^−1^ compound in order to evaluate the cell cycle distribution. After treatment period cells were taken from the substrate, fixed in 70% cold ethanol for at least 30 min at −20 °C, washed twice in PBS, and then incubated 15 min at 37 °C with RNase A (100 µg mL^−1^), and 1 h with PI (100 µg mL^−1^). After staining of cells with PI the acquisition was done using the Epics flow cytometer. Data were analyzed using FlowJo software (BD Bioscience, CA, USA) and expressed as fractions of cells in the different cell cycle phases [51].

For gene expression analysis 5 × 10^5^ HEp-2 cells/well were plated in 6-well plate and treated for 24 h with 100 µg mL^−1^ compound. After treatment the cells were taken from the substrate, and subjected to RNA extraction using RNeasy kit (Qiagen, Venlo, The Netherlands) according to the manufacturer’s protocol. The quality of isolated RNA was assessed by determining the concentration and purity using a Nanodrop spectrophotometer (Waltham, MA, USA). 2 µg RNA were mixed with 1 mM dNTP and 1.5 pmol of random primers, and incubated at 70 °C for 10 min, allowed to cool. The reverse transcription (RT) reaction carried out using 200 units Superscript reverse transcriptase (Promega, Madison, WI, USA), 1 mM DTT and 1 mMRNase inhibitor and incubation for 60 min at 37 °C and 15 min at 70 °C. The quality of cDNAs determined using PCR assay for β-2-microglobulin (B2M) house-keeping gene and agarose gel electrophoresis (2%) analysis. 50 ng mL^−1^ cDNA from each sample was used in real time PCR reaction using prevalidated Taqman Gene Expression Assays (Applied Biosystems, Foster City, CA, USA), and human beta actin as endogenous control (Applied Biosystems 4326315E). Results were analyzed with RQ study software (Applied Biosystems) [52].

## 4. Conclusions

The structure of some cobalt(II) complexes with 5,7-dimethyl-1,2,4-triazolo[1,5-*a*]pyrimidine (dmtp) have been determined by single crystal X-ray diffraction. The dmtp coordinates as unidentate through triazole nitrogen N3 and an unusual *cis* arrangement of dmtp units observed for complex **1** was correlated with the supramolecular packing of compound. Electronic spectra display the patterns of distorted octahedral or tetrahedral Co(II) coordination environments, data confirmed furthermore by the magnetic behavior of the complexes at room temperature. 

Concerning the antimicrobial activity, the results demonstrated that complex **2** exhibited the most significant antimicrobial activity against planktonic cells and the most intensive anti-biofilm effect, even at subinhibitory concentrations, having thus potential applications for the development of novel antimicrobial materials or strategies for fighting medical biofilm pathogens frequently implicated in the etiology of chronic infections.

The flow cytometry analysis revealed that one of the main mechanisms of the antimicrobial activity of the complexes is represented by the induction of lesions at the level of cellular membranes affecting their selective permeability. The higher activity of complex **2** could be explained by its lower molecular weight and non-electrolytic nature facilitating the easier crossing of the membrane.

Moreover, the complex **2** induced the apoptosis of HEp-2 cells and interfered with different molecular pathways implicated in the cellular turnover and transport, opening the perspective of their further investigation as potential anti-tumor agents. A putative mechanism for the pro-apoptotic and antimicrobial activity of these compounds is the interference with Ca^2+^ transport.

## Figures and Tables

**Table 1:** Summary of crystal data for [Co(dmtp)_2_(OH_2_)_4_][CoCl_4_] (**1**), [Co(dmtp)_2_Cl_2_] (**2**) and [Co(dmtp)_2_(OH_2_)_4_]Cl_2_·2H_2_O (**3**).

Compound	[Co(dmtp)_2_(OH_2_)_4_][CoCl_4_] (1)	[Co(dmtp)_2_Cl_2_] (2)	[Co(dmtp)_2_(OH_2_)_4_]Cl_2_·2H_2_O (3)
Color/shape	Turquoise/plate	Blue/plate	Yellow/plate
Empirical formula	C_14_H_24_Cl_4_Co_2_N_8_O_4_	C_14_H_16_Cl_2_CoN_8_	C_14_H_28_Cl_2_CoN_8_O_6_
Formula weight	628.07	426.18	534.27
Temperature	223(2) K	223(2) K	223(2) K
Crystal system	monoclinic	orthorhombic	triclinic
Space group	*C*2/c	*P*bca	P$\bar{1}$
Unit cell dimensions	*a* = 11.4180(2) *b* = 11.9909(3) Å *c* = 17.9687(4) Å β = 102.643(1)°	*a* =10.5555(1) Å *b* =18.2855(2) Å *c* =18.4002(2) Å	*a* =7.6872(4) Å *b* =8.3348(8) Å *c* =9.6388(9) Å α = 99.636(8)° β = 100.828(7)° γ = 96.026(4)°
Volume	2400.48(9) Å^3^	3551.47(6) Å^3^	592.05(9) Å^3^
Z	4	8	1
Calculated density	1.738 mg/m^3^	1.594 mg/m^3^	1.498 mg/m^3^
Absorption coefficient	1.865 mm^−1^	1.283 mm^−1^	8.169 mm^−1^
F(000)	1272	1736	277
Goodness-of-fit on F^2^	1.050	1.047	1.079
Final R indices [I > 2σ(I)]	R_1_ = 0.0366, wR_2_ = 0.0747	R_1_ = 0.0333, wR_2_ = 0.0762	R_1_ = 0.0433, wR_2_ = 0.1114
R indices (all data)	R_1_ = 0.0460, wR_2_ = 0.0804	R_1_ = 0.0411, wR_2_ = 0.0811	R_1_ = 0.0470, wR_2_ = 0.1149
Largest difference peak and hole	0.388 and −0.333 e.Å^−3^	0.364 and −0.298 e.Å^−3^	0.274 and −0.406 e.Å^−3^

**Table 2:** IR absorption bands (cm^−1^) for dmtp and complexes.

Compound				Assignments
Dmtp	(1)	(2)	(3)	
-	3543 s 3398 vs	-	3415 w3314 w	ν(OH_2_)
3101 m	3116 m	3119 w	3123 w	ν(CH)
2918 w	2950 w	2920 w	2955 w	ν_as_(CH_3_)
2833 w	2883 w	2859 w	2886 w	ν_s_(CH_3_)
1635 s 1551 vs	1627 vs 1557 vs	1625 s 1553 s	1624 s 1553 vs	ν(C=N)
1445 m	1444 m	1426 m	1441 m	ν(C=C) + δ_as_(CH_3_)
1038 w	1031 w	1040 w	1039 w	ν(N–N)
810 w	863 w	856 w	891 w	γ(CH)
-	778 w	-	779 w	ρ(H_2_O)
-	662 w	-	662 w	ρ_w_(H_2_O)
-	573 w	-	535 w	ν(Co–O)
-	492 w	487 w	489 w	ν(Co–N)

ν, stretching; δ, in plane bending; γ, out of plane bending; ρ, rocking; vs, very strong; s, strong; m, medium; w, weak; s, symmetric; as, asymmetric; w, wagging.

**Table 3:** Absorption maxima, assignments and magnetic moments for ligand and complexes.

Compound	Absorption Maxima (cm^−1^)	Assignment	Magnetic Moment (B.M.)
dmtp	37,040 33,330 27,025	π→π*	-
[Co(dmtp)_2_(OH_2_)_4_][CoCl_4_] (**1**)	37,740 34,480	π→π*	4.47
	19,400	^4^T_1g_→^4^T_1g_(P)	
	15,625 15,040	^4^A_2_→^4^T_1_(P)	
	8440	^4^T_1g_→^4^T_2g_	
	6310 6100 5500	^4^A_2_→^4^T_1_(F)	
[Co(dmtp)_2_Cl_2_] (**2**)	34,485	π→π*	4.29
	15,870 15,150	^4^A_2_→^4^T_1_(P)	
	8930 6830 5900	^4^A_2_→^4^T_1_(F)	
[Co(dmtp)_2_(OH_2_)_4_]Cl_2_∙2H_2_O (**3**)	38,460 30,765	π→π*	5.00
	20,620	^4^T_1g_→^4^T_1g_(P)	
	8625 7510	^4^T_1g_→^4^T_2g_	

**Table 4:** The complexes influence on biofilm formation (MBEC value, μg mL^−1^).

Strain	Complex		
	(1)	(2)	(3)
*E. coli* ATCC 25922	inhibition 62.50	inhibition 62.50	-
*E. coli* 832	inhibition 62.50	inhibition 250	-
*K. pneumoniae* ATCC 134202	stimulation	inhibition 62.50	-
*K. pneumoniae* 806	-	inhibition 62.50	-
*S. aureus* MRSA 1263	-	inhibition 125	-
*B. subtilis* ATCC 6633	inhibition 62.50	inhibition 125	inhibition 250
*C. albicans* ATCC 22	inhibition 62.50	inhibition 62.50	-

**Table 5:** The effects produced by the treatment of HEp-2 cells with 100 µg mL^−1^ solution of ligand and complexes for 24 h.

Species	Necrotic Cells	Dead Cells	Early Apoptotic Cells	Viable Cells
HEp-2	0.56	0.28	3.15	96.00
dmtp	2.37	2.27	3.14	92.20
(**1**)	11.30	2.85	6.98	78.90
(**2**)	7.54	16.10	20.40	55.90
(**3**)	6.19	10.80	16.90	66.20

## Supplementary Materials

The following are available online. Figure S1: IR spectra of dmtp and complexes (**1**)–(**3**), Figure S2: UV-Vis-NIR spectra of complexes , Figure S3: Experimental and simulated XRD patterns for complexes (**1**)-(**3**), Table S1: Selected bond lengths (Å) and angles (°) for complex [Co(dmtp)_2_(OH_2_)_4_][CoCl_4_] (**1**), Table S2: Intra- and intermolecular contacts (Å and °) ^a^ in compound (**1**), Table S3: Selected bond lengths (Å), angles (°) and aromatic interactions (Å) for complex [Co(dmtp)_2_Cl_2_] (**2**), Table S4: Selected bond lengths (Å) and angles (°) for complex [Co(dmtp)_2_(OH_2_)_4_]Cl_2_·2H_2_O (**3**), Table S5: Intra- and intermolecular contacts (Å and °)^a^ in compound (**3**).
