# Supplementary material for: Synthesis, Physico-chemical Characterization, Crystal Structure and Influence on Microbial and Tumor Cells of Some Co(II) Complexes with 5,7-Dimethyl-1,2,4-triazolo[1,5-a]pyrimidine
Source: Molecules. 2017 Jul 22;22(7):1233. doi: 10.3390/molecules22071233 (PMC6152184; doi:10.3390/molecules22071233)
Supplement: Supplementary file 1 [file molecules-22-01233-s001.pdf]

Supplementary materials: Synthesis, physico-chemical characterization, crystal structure and influence on microbial and tumor cells of some Co(II) complexes with 5,7-dimethyl-1,2,4-triazolo[1,5-*a*]pyrimidine

Luminiţa Măruţescu, Larisa Calu, Mariana Carmen Chifiriuc, Coralia Bleotu, Constantin-Gabriel Daniliuc, Denisa Fălcescu, Crina Maria Kamerzan, Mihaela Badea and Rodica Olar

Table S1. Selected bond lengths (Å) and angles (°) for complex $[Co(dmtp)_2(OH_2)_4][CoCl_4]$ (**1**).

Co1-O2	2.085(2)	Cl1-Co2-Cl1*	113.63(4)
Co1-O1	2.113(2)	Cl1-Co2-Cl2	108.50(2)
Co1-N3	2.154(2)	Cl1-Co2-Cl2*	108.29(3)
Co2-Cl1	2.276(1)	O1-Co1-O2	90.71(9)
Co2-Cl2	2.279(1)	O1-Co1-O1*	93.34(13)
N1-C2	1.322(4)	O1-Co1-O2*	84.10(9)
N1-N8	1.375(3)	O2-Co1-O2*	172.45(13)
N3-C2	1.356(3)	O1-Co1-N3	88.98(9)
N3-C3a	1.338(3)	O2-Co1-N3	89.93(8)
N4-C3a	1.343(3)	O1-Co1-N3*	173.62(8)
N4-C5	1.328(3)	O2-Co1-N3*	95.45(8)
N8-C7	1.367(3)	N3-Co1-N3*	89.33(11)
N8-C3a	1.370(3)		
C5-C6	1.417(3)		

*Symmetry transformations used to generate equivalent atoms: -x+1, y, -z+1/2.

Table S2. Intra- and intermolecular contacts (Å and °)[a] in compound (**1**).

D-H···*A*	*d*(*D*-H)	*d*(H···*A*)	*d*(*D*···*A*)	∠(*DHA*)
C9-H9···O1[#1]	0.97	2.619(3)	3.554	161.9
O1-H01···Cl2[#2]	0.85(4)	2.285(3)	3.105	161.1
O1-H02···Cl1[#3]	0.81(5)	2.376(2)	3.182	177.8
O2-H03···N4	0.81(5)	1.887(2)	2.730	149.9
Cg(dmtp)···Cg(dmtp)[#4,b]			3.466	

[a]Symmetry transformations used to generate equivalent atoms: [#1] –x+1, -y, -z+1; [#2] x, y+1, z; [#3] –x+1/2, y+1/2, -z+1/2; [#4] –x+1, -y, -z+1. [b]Cg is the centroid of atoms C3a/N4/C5/C6/C7/N8.

Table S3. Selected bond lengths (Å), angles (°) and aromatic interactions (Å) for complex $[Co(dmtp)_2Cl_2]$ (**2**).

Co1-N3A	2.025(2)	N1A-N8A	1.375(2)
Co1-N3B	2.040(2)	N1B-N8B	1.376(2)
Co1-Cl2	2.216(1)	N8A-C7A	1.366(2)
Co1-Cl1	2.260(1)	N8B-C7B	1.369(2)
N1A-C2A	1.316(3)	N8A-C3aA	1.368(2)
N1B-C2B	1.318(3)	N8B-C3aB	1.369(2)
N3A-C2A	1.355(2)	N4A-C3aA	1.336(2)
N3B-C2B	1.357(3)	N4B-C3aB	1.355(2)
N3A-C3aA	1.344(2)	N4A-C5A	1.333(3)
N3B-C3aB	1.343(2)	N4B-C5B	1.329(2)
Cl1-Co1-Cl2	116.11(2)	Cl2-Co1-N3A	115.00(5)
Cl1-Co1-N3A	105.12(5)	Cl2-Co1-N3B	111.51(5)
Cl1-Co1-N3B	104.22(5)	N3A-Co1-N3B	103.59(6)
π···π interactions: *C*g(dmtp)···*C*g(dmtp) 3.521[#1,a]			
C3aB···N1A 3.158[#2,b]			

Symmetry transformations used to generate equivalent atoms: #1: -x, -y+1, -z+1; #2: -x+1, -y+1, -z+1. [a]Cg is the centroid of atoms C3a/N4/C5/C6/C7/N8; [b]interactions between the dmtp ligand and the nitrogen atom from the neighboring triazol ring.

Table S4. Selected bond lengths (Å) and angles (°) for complex $[Co(dmtp)_2(OH_2)_4]Cl_2{\cdot}2H_2O$ (**3**).

Co1-O1	2.096(2)	N4-C3a	1.343(4)
Co1-O2	2.059(3)	N4-C5	1.334(4)
Co1-N3	2.151(3)	N8-C3a	1.367(4)
N1-C2	1.317(4)	N8-C7	1.373(4)
N1-N8	1.376(4)	C5-C6	1.419(5)
N3-C2	1.352(4)	C6-C7	1.362(5)
N3-C3a	1.334(4)		
O1-Co1-O2	86.21(11)	O1-Co1-N3	92.63(10)
O1-Co1-O1*	180.0	O2-Co1-N3	89.78(11)
O2-Co1-O2*	180.0	N3-Co1-N3*	180.0

*Symmetry transformations used to generate equivalent atoms: -x-1, -y, -z.

Table S5. Intra- and intermolecular contacts (Å and °)[a] in compound (**3**).

D-H···*A*	*d*(*D*-H)	*d*(H···*A*)	*d*(*D*···*A*)	∠(*D*H*A*)
O1-H1B···N4	0.78(2)	2.200	2.870	144.3
O2-H2A···Cl1	0.78(2)	2.324	3.093	168.7
O3-H3A···Cl1	0.78(2)	2.375	3.156	173.0
O1-H1A···O3[#1]	0.79(2)	1.902	2.685	173.7
O2-H2B···Cl1[#2]	0.79(2)	2.344	3.106	163.1
O3-H3B···Cl1[#3]	0.78(2)	2.523	3.270	160.3
*C*g(dmtp)···*C*g(dmtp)[b]			3.70	

[a]Symmetry transformations used to generate equivalent atoms: [#1] –x-1, -y, -z; [#2] -x, -y, -z; [#3] –x, -y+1, -z; [b]Cg is the centroid of atoms C3a/N4/C5/C6/C7/N8 from pyrimidine aromatic ring.

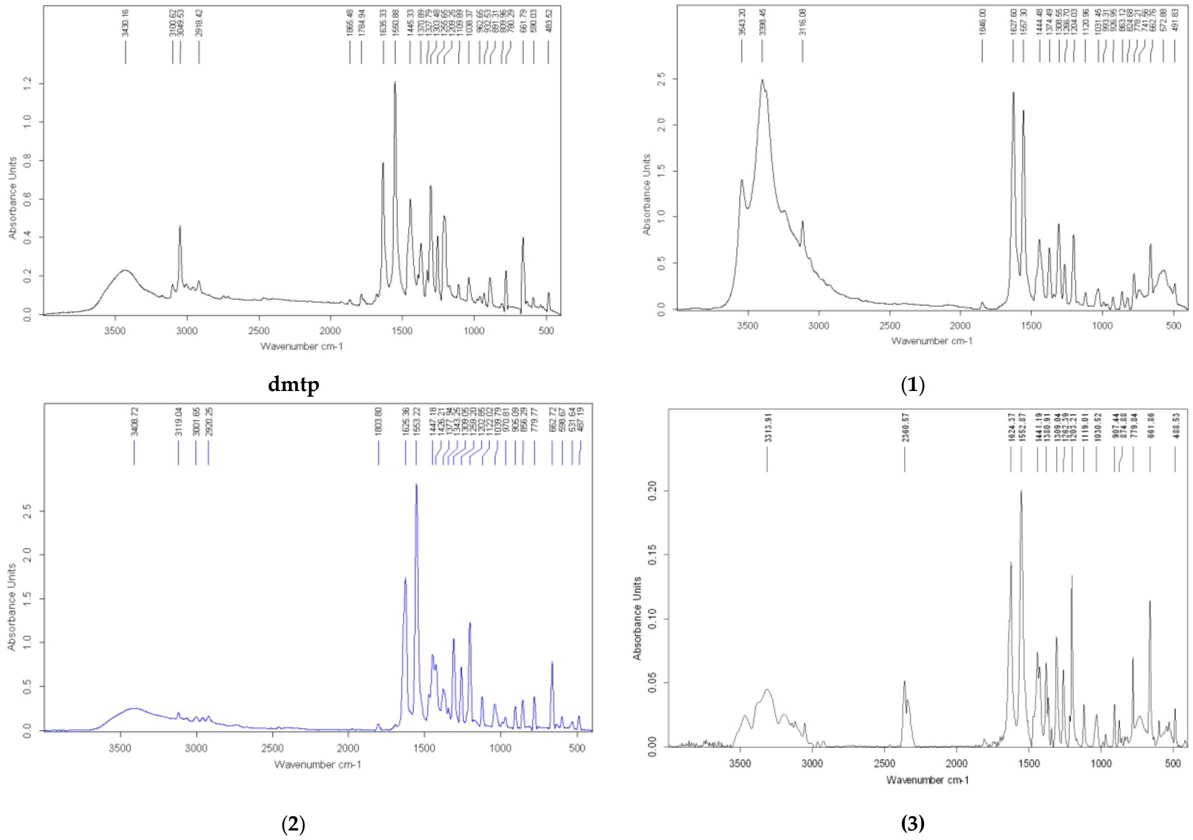

Figure S1. IR spectra of dmtp and complexes (**1**)-(**3**).

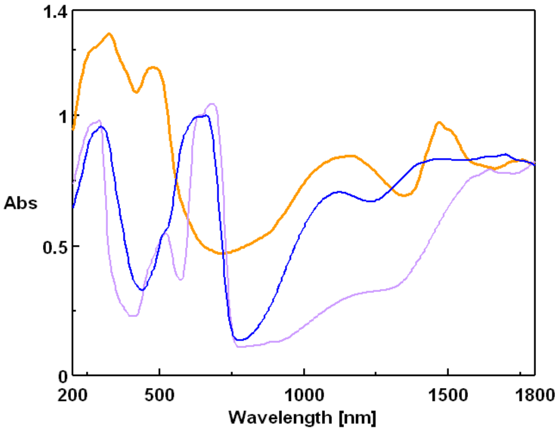

Figure S2. UV-Vis-NIR spectra of complexes: (**1**) (purple), (**2**) (blue) and (**3**) (orange).

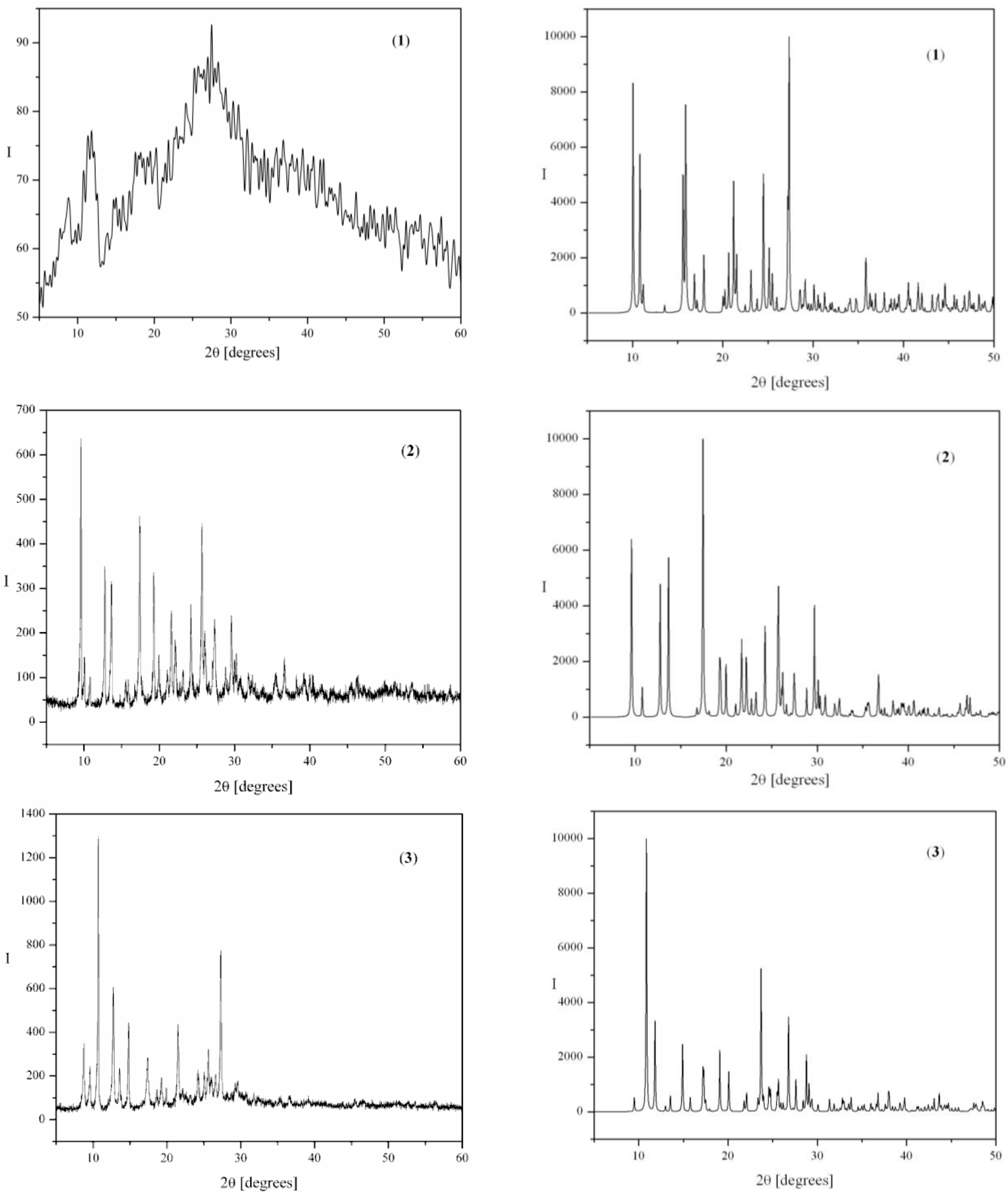

Figure S3. Experimental (left) and simulated (right) XRD patterns for complexes (**1**)-(**3**).
